# Application of Machine Learning Methods to Predict the Air Half-Lives of Persistent Organic Pollutants

**DOI:** 10.3390/molecules28227457

**Published:** 2023-11-07

**Authors:** Ying Zhang, Liangxu Xie, Dawei Zhang, Xiaojun Xu, Lei Xu

**Affiliations:** Institute of Bioinformatics and Medical Engineering, School of Electrical and Information Engineering, Jiangsu University of Technology, Changzhou 213001, China; 18726094010@163.com (Y.Z.); zdw@jsut.edu.cn (D.Z.)

**Keywords:** persistent organic pollutants, air half-life, quantitative structure–activity relationships, molecular descriptors, machine learning

## Abstract

Persistent organic pollutants (POPs) are ubiquitous and bioaccumulative, posing potential and long-term threats to human health and the ecological environment. Quantitative structure–activity relationship (QSAR) studies play a guiding role in analyzing the toxicity and environmental fate of different organic pollutants. In the current work, five molecular descriptors are utilized to construct QSAR models for predicting the mean and maximum air half-lives of POPs, including specifically the energy of the highest occupied molecular orbital (HOMO_Energy_DMol3), a component of the dipole moment along the z-axis (Dipole_Z), fragment contribution to SAscore (SAscore_Fragments), subgraph counts (SC_3_P), and structural information content (SIC). The QSAR models were achieved through the application of three machine learning methods: partial least squares (PLS), multiple linear regression (MLR), and genetic function approximation (GFA). The determination coefficients (*R*^2^) and relative errors (*RE*) for the mean air half-life of each model are 0.916 and 3.489% (PLS), 0.939 and 5.048% (MLR), 0.938 and 5.131% (GFA), respectively. Similarly, the determination coefficients (*R*^2^) and *RE* for the maximum air half-life of each model are 0.915 and 5.629% (PLS), 0.940 and 10.090% (MLR), 0.939 and 11.172% (GFA), respectively. Furthermore, the mechanisms that elucidate the significant factors impacting the air half-lives of POPs have been explored. The three regression models show good predictive and extrapolation abilities for POPs within the application domain.

## 1. Introduction

With the continuous development of the chemical industry, there are an increasing number of persistent organic pollutants (POPs) that are synthesized due to human activities. They are transmitted through specific environmental media, which not only pose potential risks to ecosystems but also pose potential threats to human health [1]. POPs are unavoidable byproducts generated during the processes of using pesticides, industrial chemicals, urban waste, wood combustion, and automobile emissions. POPs are hydrophobic organic compounds that are difficult to degrade and have low water solubility and high toxicity [2]. The severity and persistence of POP pollution have always been important issues affecting global and human health. The Seveso chemical pollution incident in Italy and the Love Canal pollution incident in the United States, both occurring in the 20th century, were environmental events caused by POP contamination. However, with the signing of the Stockholm Convention in the 21st century, countries around the world have begun to actively strengthen their management and control efforts to identify, reduce, and eliminate the release and use of POPs [3]. Due to the global nature of environmental pollution caused by POPs, it is urgently necessary to determine the physicochemical properties required for comprehensive risk assessment of POPs [4]. This will further reveal their environmental behavior, ecological risks, and bioaccumulative effects.

Performing experiments to obtain the necessary data of POPs can be a costly and time-consuming task, and handling hazardous or reactive chemicals can pose difficulties [5]. The quantitative structure–activity relationship (QSAR) is currently the most widely used computational method that predicts the biological activity, properties, and toxicity of compounds by deriving descriptors from their chemical structures [6,7]. This method is particularly suitable for dangerous, toxic, and unstable compounds. It is based on existing experimental data and utilizes techniques such as artificial intelligence, machine learning, and mathematical modeling to establish the relationship between the structural characteristics of POPs and their properties, behavior, and toxicity, thereby developing predictive models. QSAR studies have demonstrated several characteristics such as comprehensiveness, theoretical basis, intelligence, programmability, and practicality [8,9,10]. Therefore, they are considered cost-effective tools for preventing human health and environmental safety hazards caused by toxic substances [11,12,13]. Molecular fingerprint QSAR (MF-QSAR) is simpler and more efficient approach to QSAR modeling, where molecular fingerprints encode the molecular features of compounds as binary vectors, with the positions and values of the vectors representing the structural information of the compounds. Compared to molecular descriptors, QSAR models based on molecular fingerprints have advantages such as faster computation speed, smaller prediction errors, and a more comprehensive representation of molecular structures [14,15,16].

In previous literature, there have been reports on the use of various theoretical descriptors to construct QSAR models for predicting the degradation rates of POPs in air [17]. Compared to the previous studies, Khan et al. utilized the partial least squares (PLS) regression method to establish a QSAR model for the average half-life of 302 different POPs in air [7]. They employed a genetic algorithm (GA) to screen molecular descriptors and obtained the following parameter results: the optimal model consisted of six descriptors, with an *R*^2^ value of 0.663 and *Q*^2^ value of 0.640. Gramatica et al. utilized more sophisticated 3D descriptors to construct QSAR models for the air half-life of 60 POPs [17]. Although the models achieved an *R*^2^ of 0.850 and *Q*^2^ of 0.80, they did not validate the predictive quality of unknown compounds with any testing or external datasets. Zhu and Tao employed seven different machine learning methods to construct 13 quantitative structure–property relationship (QSPR) models for predicting the polydimethylsiloxane–air partition coefficient (K_PDMS-air_) [18]. They found that the gradient boosting decision tree (GBDT) model demonstrated superior predictive accuracy and interpretability, with parameter results of *R*^2^*_adj_* = 0.995 and *Q*^2^*_BOOT_* = 0.980.

In the current work, the average and maximum half-lives of 60 POPs in air were obtained from previous literature and their molecular structures are listed in Figure S1 [17]. The main differences among these pollutants are their degree of chlorination and the position of carbon–chlorine bonds [19]. To enhance the accuracy of the model predictions, six QSAR models were built to explore correlations between the mean and maximum half-life in air with selected molecular descriptors and molecular fingerprints. The model is built using PLS, genetic function approximation (GFA), and multiple linear regression (MLR) techniques. The robustness and predictive stability of the constructed models are evaluated using internal and external validation methods [20]. The resulting QSAR model provides predictive values and characterization of the outcomes.

## 2. Results

In the present study, we employed the stepwise regression (SR) method for variable selection. The SR method requires setting a significance level for variable entry and variable removal to assess the significance of the dependent variable. If a variable significantly improves the explanatory power of the model, it is included in the model. Conversely, if a variable does not contribute significantly to the model, it is removed. Ultimately, we obtained a regression model that includes the optimal combination of variables.

**Figure 1:**
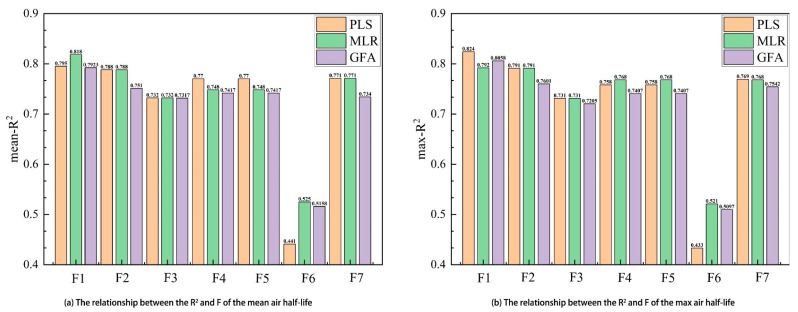
The relationship between the model’s coefficient of determination (R^2^) and different threshold values (F) by PLS, MLR, and GFA.

In the process of developing QSAR models, selecting suitable molecular descriptors can provide information about the molecular structure and properties and enhance the accuracy and stability of the model predictions. To determine the optimal combination of descriptors, we employed stepwise regression analysis to construct QSAR models for POPs with mean and maximum half-lives in air and performed analyses under different threshold conditions. Figure 1 illustrates the relationship between the model coefficient *R*^2^ and different threshold values F for three regression methods. It can be observed that when the threshold value F is between 0.05 and 0.15, the *R*^2^ values for all three regression methods reach their maximum. Specifically, PLS has an *R*^2^ value of 0.793. MLR has an *R*^2^ value of 0.818, and GFA has an *R*^2^ value of 0.792. In addition, five descriptors were identified as most closely related to the air mean and maximum half-life of POPs. Table 1 presents the selected molecular descriptors for different threshold conditions.

### 2.1. Dividing the Dataset

**Figure 2:**
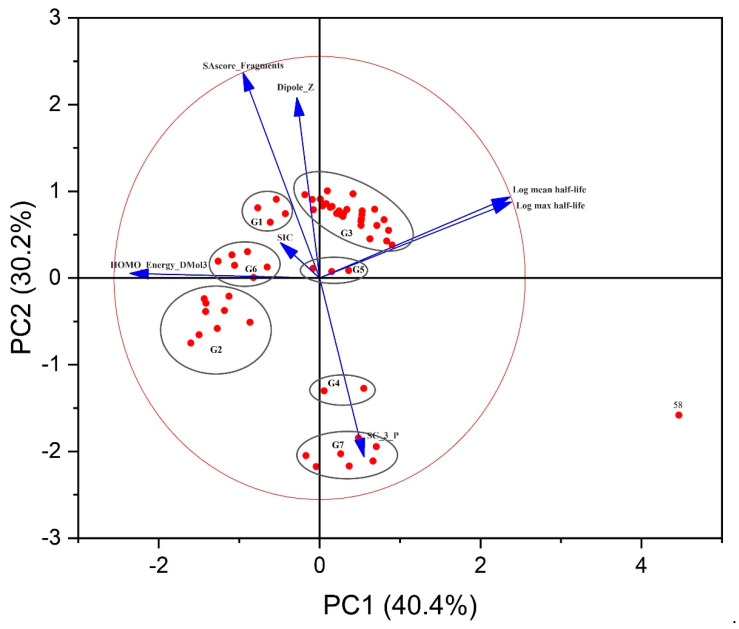
A two-dimensional plot of principal component analysis.

The structure of a molecule is responsible for its biological activity and chemical reactivity, so compounds with similar physical and chemical properties or structures tend to have similar activities [21]. We used an unsupervised learning method, principal component analysis (PCA), to classify a dataset containing 59 compounds, dividing the dataset into a training set and a test set according to the aggregation of these compounds in the principal component space with the ratio of 4:1. By observing the spatial distribution of each compound, we divided the dataset into seven categories, where G1–G7 represent cycloalkanes, tricyclic compounds, chlorinated biphenyls, hexachlorocyclohexanes, mixtures of chlorinated biphenyls and alkynes, insecticides, and dibenzofurans and their derivatives, respectively. In a two-dimensional graph, the arrows represent the principal component loadings, which reflect the correlation coefficients between the original variables and the principal components. The values of the projections of the arrows onto the coordinate axes represent the positive or negative correlation and the magnitude between the variables and the principal components. The large circles represent confidence ellipses (usually 95% confidence intervals), and the points outside the circles are not statistically significant. The small dots represent the sample points, and the distances between the lines connecting the sample points reflect the similarity between the variables. If the distances between the lines connecting the sample points of each group are short, it indicates that there is less variability between the samples. Based on the information shown in Figure 2 and Table S1, it can be observed that the cumulative variance contribution of PC1 and PC2 is 70.62%, and all eigenvalues are greater than 1. PC1 and PC2 explained 40.42% and 30.20% of the variance, respectively. Therefore, it can be concluded that the first three principal components effectively capture information on various indicators of persistent pollutants. Among them, there is a strong correlation between the mean and maximum air half-lives of POPs and the descriptor HOMO_Energy_DMol3. Two-dimensional PCA plots show the importance of each variable in the two principal components: log air half-lives (mean and max) and HOMO_Energy_DMol3 in the first principal component, while Dipole_Z, SA_score_Fragments, and SC_3_P were mainly indicated in the second principal component.

### 2.2. Developing QSAR Models

**Figure 3:**
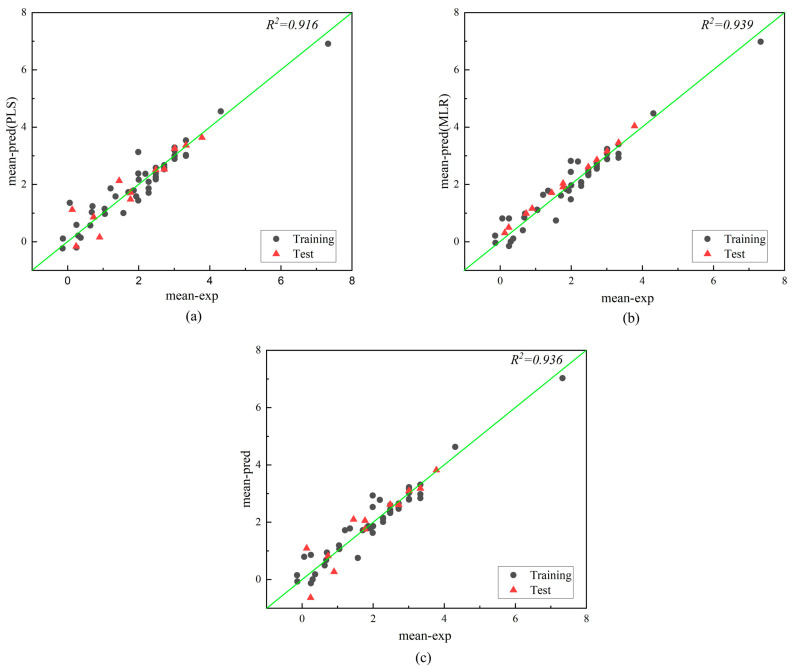
Plot of experimental and predicted values for mean air half-life modeled by PLS (**a**), MLR (**b**), and GFA (**c**).

**Figure 4:**
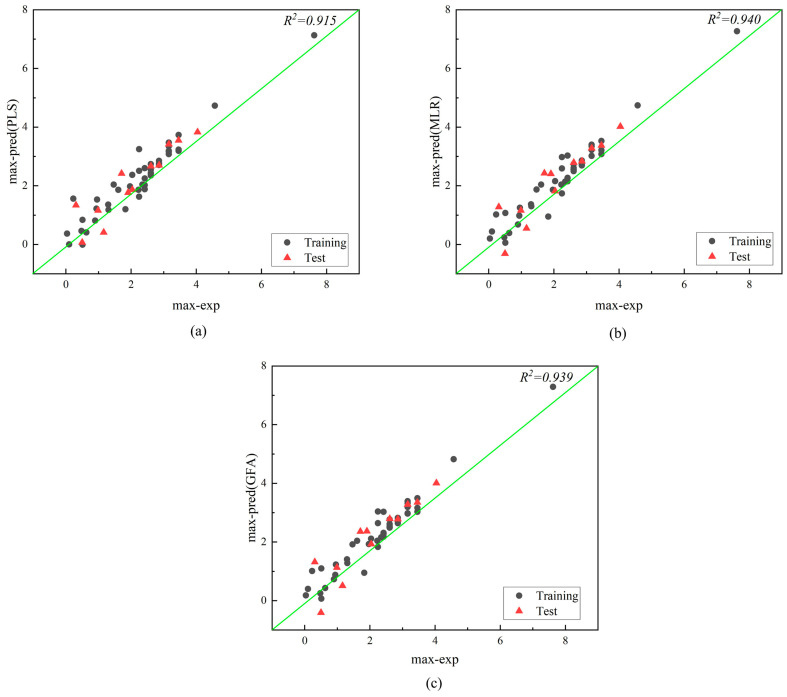
Plot of experimental and predicted values for maximum air half-life modeled by PLS (**a**), MLR (**b**), and GFA (**c**).

**Figure 5:**
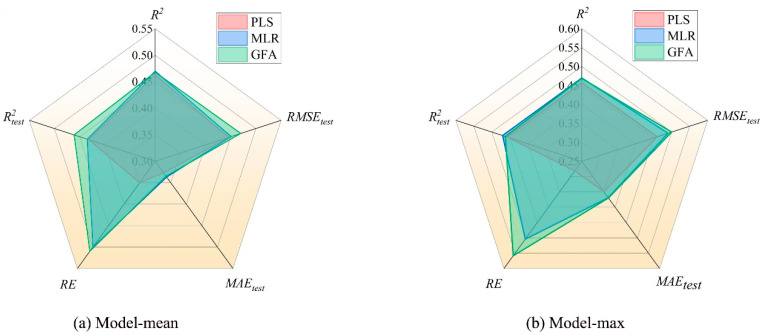
Radar model diagram of three models and five indicators (PLS, MLR, GFA) for air half-life.

We employed PLS, MLR, and GFA to establish quantitative relationship models between molecular descriptors and air half-lives. The distributions of experimental and predicted values for mean and maximum half-lives are shown in Figure 3 and Figure 4. All data points are scattered around the best-fit line, indicating the consistency between the predicted and experimental values of the constructed models and further confirming the acceptability of the models [22]. To visually represent the parameters of each model, we appropriately normalized the data and provided the metrics *R*^2^, *R*^2^*_text_*, *RE*, *MAE_test_*, and *RMSE_test_* for three regression models (Figure 5). The fitting effects, hyperparameters (Table 2), and statistical parameters (Table 3) of the three regression models are shown below:

#### 2.2.1. PLS

Log mean half-life = 2.428 + 0.3712 × Dipole_Z − 0.8169 × HOMO_Energy_DMol3 + 6.818 × SAscore_Fragments

Log max half-life = −2.239 + 0.3601 × Dipole_Z − 0.8013 × HOMO_Energy_DMol3 + 6.697 × SAscore_Fragments

#### 2.2.2. MLR

Log mean half-life = −6.271 − 24.02 × HOMO_Energy_DMol3 + 5.733 × SAscore_Fragments + 0.03767 × SC_3_P + 0.7781 × SIC

Log max half-life = −6.054 − 24.98 × HOMO_Energy_DMol3 + 5.615 × SAscore_Fragments + 0.04197 × SC_3_P + 0.4352 × SIC

#### 2.2.3. GFA

Log mean half-life = −6.5678 − 27.426 × HOMO_Energy_DMol3 + 5.7146 × SAscore_Fragments + 0.0409 × SC_3_P

Log max half-life = −6.2201 − 26.89 × HOMO_Energy_DMol3 + 5.605 × SAscore_Fragments + 0.043771 × SC_3_P

According to Table 3, the statistics of the three models show that they perform satisfactorily in terms of internal and external predictive ability. The parameters of the three models constructed in this study meet the criteria [23] (except for GFA), with *R*^2^ > 0.70 and *Q*^2^ > 0.60, and *R*^2^ − *Q*^2^ < 0.3, indicating good model fit, robustness, and predictive performance. By comparing the comprehensive performance parameters of the three models, it can be seen that the MLR model performs the best in all aspects among the three models, followed by the PLS model, and finally the GFA model. The *R*^2^ and *R*^2^*_adj_*of the MLR model are 0.931 and 0.915, respectively. *R*^2^ represents the goodness of fit to the observed values, and *R*^2^*_adj_* takes into account the influence of model complexity, which can better evaluate the predictive ability of the model. The *RMSE_test_* and *MAE_test_* values of the MLR model are all lower than the values of the other two models. This indicates that the MLR model has smaller prediction errors and more stable prediction performance. The fitting graph of the model also demonstrates a strong correlation between the experimental and predicted values in both the training and validation datasets, further confirming the good quality of the model. The MLR method was chosen to predict 10 unknown POP compounds for external validation to make our prediction results more convincing [20], and the determination coefficients (*R*^2^) and relative errors (*RE*) for half-life are 0.919 and 3.156, further indicating that our model has good predictive power (Tables S3 and S4).

## 3. Discussion

By interpreting and analyzing the molecular descriptors of the model, insights into the molecular structures related to the mean and maximum half-lives of POPs in air are provided. In this study, a total of five molecular descriptors, namely HOMO_Energy_DMol3, Dipole_Z, SAscore_Fragments, SC_3_P, and SIC, were selected to build QSAR models based on PLS, MLR, and GFA. According to the regression equation of the model, it can be inferred that the descriptors HOMO_Energy_DMol3 and SAscore_Fragments have significant contributions to the prediction of the air half-life of POPs. From the regression coefficient in the formula, it can be seen that the values of the mean and maximum half-lives of air increase with the values of Dipole_Z, SAscore_Fragments, SC_3_P, and SIC and decrease with the value of HOMO_Energy_DMol3.

HOMO_Energy_DMol3 is a molecular descriptor that utilizes the DMol3 code to measure the highest occupied molecular orbital energy level of a molecule. HOMO represents the highest occupied molecular orbital and is used to determine the energy level of molecular orbitals as an indicator of electron-releasing capacity. Certain chlorinated organic compounds, such as DDT, DDE, and DDD, have lower HOMO energies. Since they are chlorinated compounds with high electronegativity, they attract surrounding electrons and reduce their electron-giving ability, making them less reactive and susceptible to electron transfer reactions, thus affecting the degradation rate of the compounds. On the contrary, some compounds that do not contain chlorine atoms, such as acenaphthene and dibenz[a,h]anthracene, have higher HOMO energies. These compounds contain only carbon, hydrogen, and other atoms and therefore have fewer electrons in the electron cloud. They are relatively more reactive and are more readily degraded or oxidized, thus shortening the degradation half-life of the compounds in air.

Dipole_Z is used to measure the dipole nature of a compound. Due to the geometry of the molecule and the inhomogeneity of the distribution of the electron cloud, a partial separation of positive and negative charges between the carbon and chlorine atoms occurs, forming a complex dipole. When the position of the carbon–chlorine bond is closer to the center of the molecule, the compound has a smaller difference in electronegativity, a more uniform charge distribution within the molecule, and a lower dipole. On the contrary, when the position of the carbon–chlorine bond is closer to the end of the molecule, the compound has a larger difference in electronegativity, a higher energy of chemical bonding, and a higher dipole. In general, organic pollutants with higher Dipole_Z values will have the carbon–chlorine bond position closer to the center of the molecule, which will be more easily protected by the surrounding groups. The compounds will be in a more stable state and therefore may be more difficult to degrade. Organic pollutants with lower Dipole_Z values have carbon and chlorine bonds closer to the end of the molecule, which are more susceptible to photolysis, oxidation, etc. and form larger dipole molecules, resulting in an uneven distribution of charge and a larger dipole moment of the compounds, which exacerbate the degradation of POPs.

SAscore_Fragments, on the other hand, is a molecular synthetic accessibility score based on fragment contributions that describe the surface area of the molecule at a macroscopic level and reflect the exposure of the molecule to the environment. The main definition of the score is based on the similarity to structural features observed in the PubChem subset, as well as estimates of synthetic accessibility to unusual loops and many stereocenters. Its value generally lies between 1 and 10, and the molecules with a high SAscore are more difficult to synthesize, while molecules with low SAscore values are easier to synthesize. The synthesis of compounds is subject to some variability and complexity due to various factors, including reaction conditions, reaction by-products, and chemical stability. For example, 2,2’,3,4,5-pentachlorobiphenyl contains more chlorine atoms and requires multiple chlorination reactions, which may produce some non-target products and increase the difficulty of reaction and purification. In contrast, 3-chlorobiphenyl contains only one chlorine atom, and its molecular structure is simpler and relatively unstable, so it is easier to carry out the chlorination reaction. Therefore, the synthesis of 2,2’,3,4,5-pentachlorobiphenyl was relatively more difficult than the synthesis of 3-chlorobiphenyl. It was proven that the SAscore value of 2,2’,3,4,5-pentachlorobiphenyl (0.81594) was significantly higher than the SAscore value of 3-chlorobiphenyl (0.64936), and the air average and maximum half-life values of 2,2’,3,4,5-pentachlorobiphenyl were higher than those of 3-chlorobiphenyl.

SC_3_P is a descriptor for the number and type of structural units in a molecule, which has an important influence on the properties and reactivity of the molecule. A larger value of SC_3_P indicates that the compound has a more diffuse molecular shape, a larger number of structural units, and a higher degree of volatility and lipophilicity, which results in a shorter degradation of the POPs in the atmosphere. DDT, DDE, and DDD, for example, are organochlorine compounds with a single structure (containing only benzene rings and chlorine atoms) and a regular molecular shape that are mainly removed from the atmosphere by gas-phase wet deposition and rainfall. In contrast, acenaphthene and dibenz[a,h]anthracene are polycyclic aromatic hydrocarbon compounds with flat structures and conjugated systems, which are less stable and photostable, more susceptible to photochemical and oxidative reactions, and therefore have faster degradation rates in air. It should be noted that although the SC_3_P values of DDT, DDE, and DDD were 0.50923, 0.52037, and 0.57366, respectively, which were significantly lower than the SC_3_P values of acenaphthene (0.76754) and dibenz[a,h]anthracene (0.65625), acenaphthene and dibenz[a,h]anthracene had smaller air mean and maximum half-life values than DDT, DDE, and DDD.

SIC is a metric used to characterize the structural complexity of a compound. The structural complexity of a compound is evaluated by considering several factors, such as the number and type of chemical bonds as well as the arrangement of atoms in the molecule. A higher SIC value indicates a more complex structure of the compound. For example, DDT, DDE, and DDD are larger, single molecules with relatively looser spaces and fewer chemical bonds within the molecule. As a result, they are more susceptible to attack and decomposition by oxygen, water, or other chemicals in the environment. In contrast, acenaphthene and dibenz[a,h]anthracene are large molecules composed of multiple aromatic rings. Due to the smaller space inside the molecule, the atoms in the molecule are close to each other, possessing more chemical bonds and ways of connecting the atoms. Therefore, they are relatively stable and difficult to decompose in the environment. In summary, higher SIC values are usually associated with slower and more difficult degradation of compounds in air.

**Figure 6:**
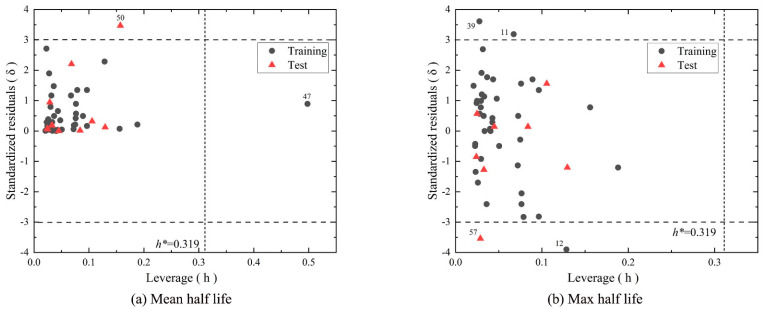
The Williams plot of the mean and maximum half-lives in air by MLR.

According to the OECD guidelines, a practical QSAR model should have a clear applicability domain (AD) [24]. In this study, we used the leverage value to quantitatively measure the extent of extrapolation and evaluate the applicability range of the model. According to Figure 6a, most of the compounds in the training set and validation set are within an acceptable range, except for the dieldrin and p, p’-DDE compounds. This indicates that the training data for the model are representative. From Figure 6b, it can be observed that there are a total of four substances in the dataset with δ > 3, which are defined as outliers. Among them, the training set contains three compounds (2,3’,4,4’-tetrachlorobiphenyl, benzo[b]fluoranthene, and benzo[a]pyrene) with δ > 3, indicating that these substances have a significant impact on the construction of the model. Furthermore, the test set includes one compound (endrin) with h < h* and δ > 3, suggesting that the structure of these two compounds significantly differs from the compounds in the training set used to build the model.

## 4. Materials and Methods 

### 4.1. Data Collection and Partitioning

In order to make the experimental data conform to a normal distribution [25,26,27,28], the logarithmic numerical forms of the air mean half-life and maximum half-life of the 60 POPs were collected from previous studies (Table 4) [17]. The air mean half-life values range from −0.14 to 7.33 and the air maximum half-life values range from 0.04 to 7.62. The 60 POPs include polycyclic aromatic hydrocarbons (PAHs), polychlorinated biphenyls (PCBs), halogenated benzenes (HBs), dioxins, furans, and pesticides. The atmospheric half-life, which is the time required for the content of POPs to decrease to half of the initial content after entering the atmosphere, was used to study the degree of degradation and dispersion pattern of these pollutants and is important for assessing their ecological risks. The division of the dataset into training and test sets is an important step in performing QSAR modeling. The training set is used for model development, while the test set compounds are used for model validation [29]. Based on OECD guidelines and rules of thumb, the entire dataset is usually randomly divided into a training set (containing 47 compounds, with mirex excluded for its high impact on the model) and a test set (containing 12 compounds) in a certain ratio (usually 4:1). However, it is difficult to ensure the diversity of compounds in the training set using the randomized division method, and thus there is some uncertainty. Therefore, in order to improve the generalization ability and prediction performance of the model, this study introduces suitable molecular descriptors by setting different threshold ranges, and then randomly divides the training set and test set according to the ratio of 4:1. Figure 3 shows the comparison results of the correlation coefficient *R*^2^ of the QSAR models constructed by the three regression methods under different threshold ranges. It is found that the *R*^2^ values of the three QSAR models are the largest in the range of threshold F from 0.05 to 0.15, which initially indicates that the models are better fitted. Finally, we selected the molecular descriptors introduced with the threshold F in the range of 0.05 to 0.15 and used PCA to convert the properties of the compounds (molecular descriptors and half-life properties) into a set of principal component scores. The aggregation of the compounds on the spatial axes of the principal components can be used to compare the similarity between different compounds, thus identifying compounds with similar properties. Therefore, not only was the best combination of molecular descriptors selected in this study but the diversity of the training and test sets was also ensured. 

### 4.2. Calculation and Filtering of Molecular Descriptors

Molecular descriptors are properties of chemical structures that are measured experimentally or derived theoretically [30]. They are highly related to the fitting performance and predictive ability, so the selection of molecular descriptors is crucial in QSAR model research. When selecting molecular descriptors, the following principles should be followed: (1) the chosen molecular descriptors should have interpretable physical and chemical significance and be relevant to the research question; (2) the chosen molecular descriptors should be as diverse as possible to cover multiple aspects of molecular structure and properties; (3) the chosen molecular descriptors should be associated with the target property or activity; (4) the chosen molecular descriptors should be computationally stable and have good repeatability.

**Figure 7:**
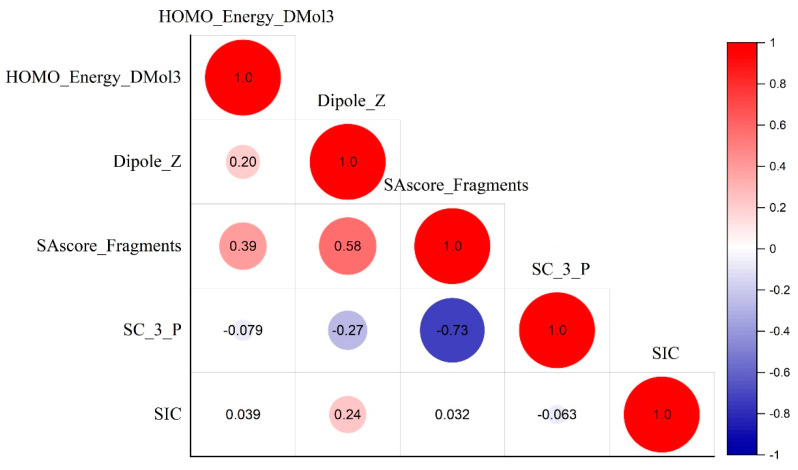
Pearson correlation of the five descriptors in this study.

First, simplified molecular input line entry system (SMILES) structures for 60 POPs were retrieved from PubChem (https://pubchem.ncbi.nlm.nih.gov (accessed on 23 November 2022)) [31]. Second, the chemical structures of 60 POPs were built with Maestro and processed with Ligprep in Schrödinger. Discovery Studio (DS) 3.5. software was then used to calculate approximately 586 molecular descriptors. These descriptors included about 10 2D, 3D, structural, thermodynamic, topological, and spatial descriptors [32]. Finally, the construction of the QSAR model was continued using the PLS, MLR, and GFA regression methods built into the commercial DS 3.5 molecular simulation software. For descriptors, we removed constant terms or descriptors that were close to constant and processed descriptors with missing values and outliers. To reduce redundancy in the descriptor data matrix [33], we used the stepwise multiple regression method to eliminate some collinear descriptors. The selected molecular descriptors should meet the inclusion threshold of F = 0.05 and exclude the threshold of F = 0.15. Additionally, the variance inflation factor (VIF) should be less than 10, and the significance level (p) should be less than 0.001. Finally, a Pearson correlation analysis was conducted by using Origin 2020 software. Following the specified requirements, five optimal descriptors were obtained. There is a good correlation between these parameters and the half-life of POPs [34]. The meanings of each descriptor are shown in Table 5. The correlation analysis among the five descriptors is illustrated in Figure 7, and the descriptor correlation matrix is presented in Table S2.

ECFP is an advanced chemical fingerprinting technique that can rapidly characterize the structural features of molecules, regardless of their specific molecular family [35]. This technique utilizes standardized resources and is capable of evaluating an individual’s exposure level by detecting the metabolites of various pollutants in the body. It achieves this by transforming the structural features of molecules into a series of bit strings. Molecular fingerprints can provide a better representation of the functional groups, chemical bonds, and chemical structures of compounds, making it easier to explain the chemical properties of compounds. To improve the quality of the prediction model, this study further introduces molecular fingerprints based on the use of molecular descriptors. Figure S2 shows the five typical molecular fingerprint patterns selected in this study.

### 4.3. Machine Learning Methods

Due to the presence of multicollinearity between the sets of independent variables and dependent variables, PLS was used to establish a regression model. PLS is a modified version of PCA regression that can analyze data with missing values, strong collinearity, noise, and numerous predictor variables [36]. PLS can identify the molecular descriptors that have a high contribution to the atmospheric half-lives of POPs and can also assist in establishing regression equations and models for these descriptors.

(1) $$T_{m}=XX^{T}Y{\left(U_{\left(m-1\right)}^{T}X^{T}Y\right)}^{\left(-1\right)}$$

(2) $$U_{m}=YXTY{\left(T_{\left(m-1\right)}^{T}X^{T}Y\right)}^{\left(-1\right)}$$

where *X* is a matrix of size *n* × *p*, *n* is the number of samples, and *p* is the number of independent variables. *Y* is a matrix of size *n* × *q*, and *q* is the number of dependent variables. PLS aims to find a new set of synthetic variables such that the covariance between them is maximized. *T* can explain the change in *X* and *Y* well.

MLR is a widely used statistical method that applies to situations involving multiple input variables. Compared to simple linear regression, MLR has a more complex structure and provides a more direct interpretation [37,38]. In MLR, we can incorporate multiple explanatory variables to measure the explanation of individuals in different aspects across multiple dimensions. The main purpose is to use the relevant model for interpretation and prediction to gain deeper insights and understanding. Thus, MLR is a powerful tool that provides us with a reliable means to solve practical problems and achieve research outcomes.

(3) Y=Xβ+ε

where *Y* is an *n* × 1 column vector with n being the number of samples, representing the observations of the dependent variable *Y*; *X* is an *n* × (*p* + 1) matrix with p being the number of independent variables; *β* is a (*p* + 1) × 1 column vector representing the regression coefficients; and *ε* is an n × 1 column vector representing the error terms.

GFA is a combination algorithm that combines a genetic algorithm (GA) with multiple adaptive regression (MAR), and it can optimize parameters. GFA identifies several independent variables that have a strong correlation with the dependent variable from a large number of independent variables. The quality of the model is evaluated using evaluation methods such as LOF. The higher the numerical value, the higher the model score [39].

(4) $$LOF=\frac{SSE}{{\left\{1-\left[c+\left(\frac{dp}{n}\right)\right]\right\}}^{2}}$$

where *SSE* is the standard error; *c* is the number of GFA models; *d* is the smoothing parameter of the model; *p* is the number of eigenvalues in the model; *n* is the number of compounds in the training set.

### 4.4. Model Evaluation and Validation

In this study, we evaluate the goodness of fit, stability, and predictive ability of the QSAR model using modeling statistical parameters and internal and external validation metrics [40,41,42]. The stability of the model under interference was evaluated using leave-one-out cross-validation (LOO_CV_). Leave-one-out cross-validation involves repeatedly training the model on the remaining samples after leaving out one sample from the training set and then uses the trained model to predict the properties of the left-out sample. The internal validation metrics for the regression model are as follows:

*R*^2^ ranges from 0 to 1. The closer *R*^2^ is to 1, the more information about the dependent variable the model can explain.

(5) $$R=\sqrt{1-\frac{\sum {\left(Y_{exp}-Y_{pred}\right)}^{2}}{\sum {\left(Y_{exp}-Y_{mean}\right)}^{2}}}$$

*RMSE* indicates the dispersion of the random error.

(6) $$RMSE=\sqrt{\frac{\sum {\left(Y_{exp}-Y_{pred}\right)}^{2}}{N}}$$

*MAE* is used to measure the error between the predicted and experimental values.

(7) $$MAE=\frac{\sum {\left|Y_{exp}-Y_{pred}\right|}^{2}}{N}$$

*Q*^2^*_CV_* is the value of the cross-validation of the extraction method, which reflects the predictive power of the QSAR model.

(8) $$Q_{cv}^{2}=1-\frac{\sum {\left(Y_{exp}-Y_{pred}\right)}^{2}}{\sum {\left(Y_{exp}-Y_{mean}\right)}^{2}}$$

*RE* is the relative error.

(9) $$RE=\frac{1}{N}\left\{\sum \left|\frac{Y_{exp}-Y_{pred}}{Y_{exp}}\right|\right\}\times 100\%$$

where *Y_exp_* and *Y_pred_* denote the experimental values and the predicted values of the models. *Y_mean_* denotes the mean of the experimental values of the compounds, and *N* denotes the number of compounds in the model

External validation is a crucial and effective step in evaluating the predictive capability of the model. It involves checking the prediction results and the actual results of molecules in the test set using the QSAR model that was built using the training set. This helps further validate the true predictive ability of the model. Common validation parameters include *Q*^2^*_ext_*.

(10) $$Q_{ext}^{2}=1-\frac{\sum {\left(X_{exp}-X_{pred}\right)}^{2}}{\sum {\left(X_{exp}-X_{mean}\right)}^{2}}$$

where the *X_exp_*, *X_pred_*, and *X_mean_* denote the experimental, predicted, and mean values of the prediction set, respectively.

According to the OECD guidelines, application domain characterization is an important part of assessing the reliability and accuracy of the model to determine the range of applicability of the model. Standard residuals (*δ*), leverage values (*h*), and Williams plots were used for model application domain characterization. *h* and *δ* were calculated as follows:

(11) $$h_{i}=x_{i}{\left(X^{T}X\right)}^{\left(-1\right)}x_{i}$$

(12) $$h^{*}=3(m+1)/n_{tr}$$

(13) $$\;\;\delta =\frac{y_{expi}-y_{predi}}{\sqrt{\frac{\sum_{i=1}^{n}{\left(y_{expi}-y_{predi}\right)}^{2}}{n-k-1}}}$$

where *x_i_* denotes the number of the *i*th compound descriptor, *X* denotes the matrix composed of compound descriptors. *m* denotes the number of molecular descriptors in the model; *n_tr_* denotes the number of training sets; *y_expi_* denotes the experimental values of compounds; and *y_predi_* denotes the predicted values of compounds.

## 5. Conclusions

This study utilized a dataset consisting of 60 organic chemical substances, with 47 substances used for model training and the remaining substances used for evaluating the predictive ability of the model. Three regression methods, namely PLS, MLR, and GFA, were employed to predict the average and maximum half-lives of POPs in air. QSAR is a method that uses the chemical structure information of substances to provide an alternative approach for experimental testing. It offers theoretical guidance for understanding the migration mechanisms of organic pollutants and evaluating their behavior in the environment. The internal and external validation of three models, namely MLR, GFA, and PLS, indicate that the MLR model has the best stability and extrapolation ability, followed by GFA and PLS. These models ultimately achieve good predictive performance and provide important references for future in-depth research.

## Figures and Tables

**Table 1:** Molecular descriptors at different thresholds under three regression methods.

	Molecular Structure Descriptors
F1 = 0.05–0.15	HOMO_Energy_DMol3, Dipole_Z, SAscore_Fragments, SC_3_P, SIC
F2 = 0.15–0.3	HOMO_Energy_DMol3, ES_Sum_aasC, SIC, Num_AtomClasses, Jurs_FNSA_3
F3 = 0.3–0.6	Num_RingFusionBonds, Jurs_FNSA_3, ES_Sum_aasC, HOMO_Energy_DMol3
F4 = 0.6–0.7	HOMO_Energy_DMol3, ES_Sum_aasC, Jurs_FNSA_3, SC_3_P, ALogP98
F5 = 0.7–0.8	HOMO_Energy_DMol3, ES_Sum_aasC, Jurs_FNSA_3, SC_3_P, ALogP98
F6 = 0.8–0.9	Jurs_FNSA_3, Num_RingFusionBonds, SC_3_P, ALogP98
F7 = 0.9–0.99	ALogP98, HOMO_Energy_DMol3, Num_AtomClasses, Jurs_FNSA_3, BIC

**Table 2:** Hyperparametric indicators for the three regression models PLS, MLR, and GFA.

Model	Standard
PLS	Cross-validation = five folds; Maximum correlation = 0.7; Dynamic smoothing factor = 0.5; Number of nearest neighbors = 20.
MLR	Cross-validation = five folds; Maximum correlation = 0.7; Dynamic smoothing factor = 0.5; Number of nearest neighbors = 20.
GFA	The number in the population = 100; The number of iterations = 50,000; Model form = linear; Maximum correlation = 0.7; LOF smoothness parameter = 0.5.

**Table 3:** Comparison of parameters between the training set and test set in three regression models.

Log Mean							
Model	*R* ^2^	*R* ^2^ *_text_*	*R* ^2^ *_adj_*	*Q* ^2^ *_cv_*	*RMSEt_est_*	*MAE_test_*	*RE* (%)
PLS	0.916	0.863	0.906	0.799	0.446	0.327	3.489
MLR	0.939	0.870	0.915	0.833	0.451	0.337	5.048
GFA	0.936	0.921	0.9006	/	0.470	0.335	5.131
**Log Max**							
**Model**	***R* ^2^**	***R* ^2^ *_text_***	***R* ^2^ *_adj_***	***Q* ^2^ *_cv_***	***RMSE_test_***	***MAE_test_***	***RE* (%)**
PLS	0.915	0.915	0.906	0.776	0.460	0.349	5.629
MLR	0.940	0.940	0.915	0.816	0.490	0.369	10.090
GFA	0.939	0.925	0.9026	/	0.500	0.370	11.172

**Table 4:** Comparison of three regression methods for air half-life data.

Name	Log Air Half-Life Values (h)							
	Mean-Exp	Mean-Pred			Max-Exp	Max-Pred		
		PLS	MLR	GFA		PLS	MLR	GFA
Naphtalene	1.21	1.86	1.64	1.72	1.47	2.04	1.87	1.92
Acenaphthene	0.68	1.03	0.85	0.68	0.94	1.22	0.98	0.88
Acenaphthylene	−0.14	−0.23	0.21	0.15	0.10	0.00	0.44	0.40
Fluorene	1.57	1.00	0.74	0.75	1.83	1.20	0.95	0.95
Anthracene	0.06	1.36	0.81	0.79	0.23	1.56	1.02	1.01
Phenanthrene	1.04	1.15	1.12	1.19	1.30	1.36	1.37	1.41
Fluoranthene	1.05	0.97	1.11	1.06	1.31	1.18	1.30	1.28
Pyrene	0.13	1.12	1.03	1.09	0.31	1.34	1.28	1.32
Chrysene	0.64	0.57	0.40	0.49	0.90	0.81	0.68	0.73
Benz[a]anthracene	0.30	0.21	0.00	0.00	0.48	0.46	0.24	0.25
Benzo[b]fluoranthene	0.90	0.16	0.35	0.27	1.16	0.41	0.55	0.51
Benzo[a]pyrene	−0.13	0.11	−0.04	−0.07	0.04	0.37	0.20	0.18
7,12-Dimethylbenz[a]anthracene	0.25	−0.20	−0.15	−0.13	0.51	−0.01	0.06	0.07
3-Methylcholanthrene	0.24	−0.16	−0.45	−0.63	0.50	0.07	−0.31	−0.41
Benzo[ghi]perylene	0.25	0.59	0.81	0.86	0.51	0.84	1.07	1.10
Dibenz[a,h]anthracene	0.37	0.14	0.11	0.18	0.63	0.41	0.39	0.43
Biphenyl	1.77	1.72	1.56	1.76	2.04	1.87	1.82	1.93
2-Chlorobiphenyl	2.28	1.71	1.95	2.01	2.42	1.88	2.15	2.18
3-Chlorobiphenyl	2.28	1.86	2.08	2.12	2.42	2.02	2.26	2.28
4-Chlorobiphenyl	2.28	2.09	2.06	2.15	2.42	2.25	2.27	2.31
2,2’-Dichlorobiphenyl	2.48	2.38	2.56	2.60	2.61	2.55	2.74	2.77
2,4-Dichlorobiphenyl	2.48	2.27	2.41	2.34	2.61	2.44	2.55	2.51
2,5-dichlorobiphenyl	2.48	2.18	2.43	2.36	2.61	2.35	2.57	2.53
3,3’-dichlorobiphenyl	2.48	2.51	2.64	2.62	2.61	2.67	2.79	2.79
3,4-Dichlorobiphenyl	2.48	2.26	2.34	2.32	2.61	2.42	2.50	2.49
3,5-Dichlorobiphenyl	2.48	2.19	2.45	2.44	2.61	2.36	2.61	2.60
4,4’-Dichlorobiphenyl	2.48	2.58	2.32	2.44	2.61	2.74	2.54	2.61
2,2’,5-Trichlorobiphenyl	2.72	2.57	2.55	2.47	2.86	2.75	2.69	2.64
2,3’,5-Trichlorobiphenyl	2.72	2.57	2.73	2.65	2.86	2.74	2.86	2.82
2,4,4’-Trichlorobiphenyl	2.72	2.67	2.57	2.54	2.86	2.85	2.73	2.71
2,4,5-Trichlorobiphenyl	2.72	2.53	2.64	2.57	2.86	2.71	2.79	2.75
2,4,6-Trichlorobiphenyl	2.72	2.53	2.71	2.61	2.86	2.70	2.84	2.78
2,3’,4’-Trichlorobiphenyl	2.72	2.60	2.64	2.58	2.86	2.77	2.78	2.75
2,2’,3,3’-Tetrachlorobiphenyl	3.01	3.01	3.24	3.22	3.16	3.19	3.40	3.39
2,2’,4,4’-Tetrachlorobiphenyl	3.01	3.29	3.21	3.16	3.16	3.47	3.35	3.32
2,2’,5,5’-Tetrachlorobiphenyl	3.01	3.18	3.07	3.02	3.16	3.35	3.22	3.19
2,2’,5,6’-Tetrachlorobiphenyl	3.01	2.89	2.89	2.79	3.16	3.08	3.02	2.97
2,2’,6,6’-Tetrachlorobiphenyl	3.01	3.25	3.11	3.10	3.16	3.42	3.28	3.28
2,3’,4,4’-Tetrachlorobiphenyl	3.01	2.98	2.88	2.81	3.16	3.16	3.02	2.98
2,2’,3,4,5-Pentachlorobiphenyl	3.33	3.00	3.07	2.98	3.46	3.19	3.21	3.16
2,2’,3,4,5’-Pentachlorobiphenyl	3.33	3.03	2.93	2.84	3.46	3.23	3.08	3.03
2,2’,4,5,5’-Pentachlorobiphenyl	3.33	3.37	3.24	3.18	3.46	3.55	3.38	3.35
2,2’,4,6,6’-Pentachlorobiphenyl	3.33	3.54	3.40	3.31	3.46	3.73	3.53	3.49
Alpha-hexachlorocyclohexane	1.71	1.73	1.61	1.72	1.97	1.98	1.86	1.93
Gamma-hexachlorocyclohexane	1.71	1.73	1.61	1.72	1.97	1.98	1.86	1.93
p,p’-DDT	1.99	3.13	2.82	2.93	2.25	3.25	2.98	3.04
p,p’-DDE	1.99	1.44	1.48	1.63	2.25	1.63	1.74	1.83
p,p’-DDD	1.99	2.38	2.44	2.53	2.25	2.51	2.59	2.64
Chlordane	1.45	2.13	2.24	2.10	1.71	2.42	2.43	2.36
Dieldrin	1.35	1.58	1.78	1.78	1.61	1.86	2.04	2.04
2,3,7,8-Tetrachloro-dibenzo-p-dioxin	1.86	1.79	1.83	1.86	2.35	2.04	2.14	2.16
1,2,3,4,7,8-Hexachloro-dibenzo-p-dioxin	1.77	1.48	2.13	2.06	1.91	1.77	2.41	2.37
Pentachlorobenzene	3.78	3.64	3.83	3.82	4.04	3.83	4.02	4.01
Hexachlorobenzene	4.31	4.55	4.48	4.63	4.57	4.73	4.74	4.82
2,3,7,8-Tetrachloro-dibenzofuran	2.19	2.37	2.80	2.78	2.42	2.60	3.03	3.03
Aldrin	0.70	1.24	0.98	0.94	0.96	1.53	1.25	1.23
Endrin	1.93	1.58	1.78	1.78	2.23	1.86	2.04	2.04
Mirex	7.33	6.91	6.98	7.03	7.62	7.13	7.27	7.29
Toxaphene	2.00	2.17	1.97	1.86	2.04	2.37	2.16	2.10
Heptachloro	0.73	0.86	0.86	0.82	0.99	1.16	1.16	1.13

**Table 5:** The names and meanings of the five descriptors used in this study.

Species	Designation	Description
Molecular descriptors	HOMO_Energy_DMol3	The energy of the highest occupied molecular orbital
	Dipole_Z	Component of the dipole moment along the z-axis
	SAscore_Fragments	Fragment contribution to SAscore
	SC_3_P	Subgraph counts
	SIC	Structural information content. Graph-theoretical info content descriptor which differentiates molecules according to their size, degree of branching, and flexibility

## Data Availability

The data presented in this study are available on request from the corresponding author.

## Supplementary Materials

The following supporting information can be downloaded at: https://www.mdpi.com/article/10.3390/molecules28227457/s1. Figure S1. Chemical structures of 60 persistent organic pollutants (POPs) used in this study; Figure S2. List several kinds of molecular fingerprints used in this study; Table S1. Features of principal component analysis; Table S2. The correlation matrix of the five descriptors used in this study; Table S3. Prediction of additional POPs by MLR modeling; Table S4. MLR model parameters for additional POPs.
