# Supplementary material for: Application of Machine Learning Methods to Predict the Air Half-Lives of Persistent Organic Pollutants
Source: Molecules. 2023 Nov 7;28(22):7457. doi: 10.3390/molecules28227457 (PMC10673120; doi:10.3390/molecules28227457)
Supplement: Supplementary file 1 [file molecules-28-07457-s001.zip › molecules-2676823-SI.pdf]

Supporting information

Figure S1 Chemical structures of 60 persistent organic pollutants (POPs) used in this study.

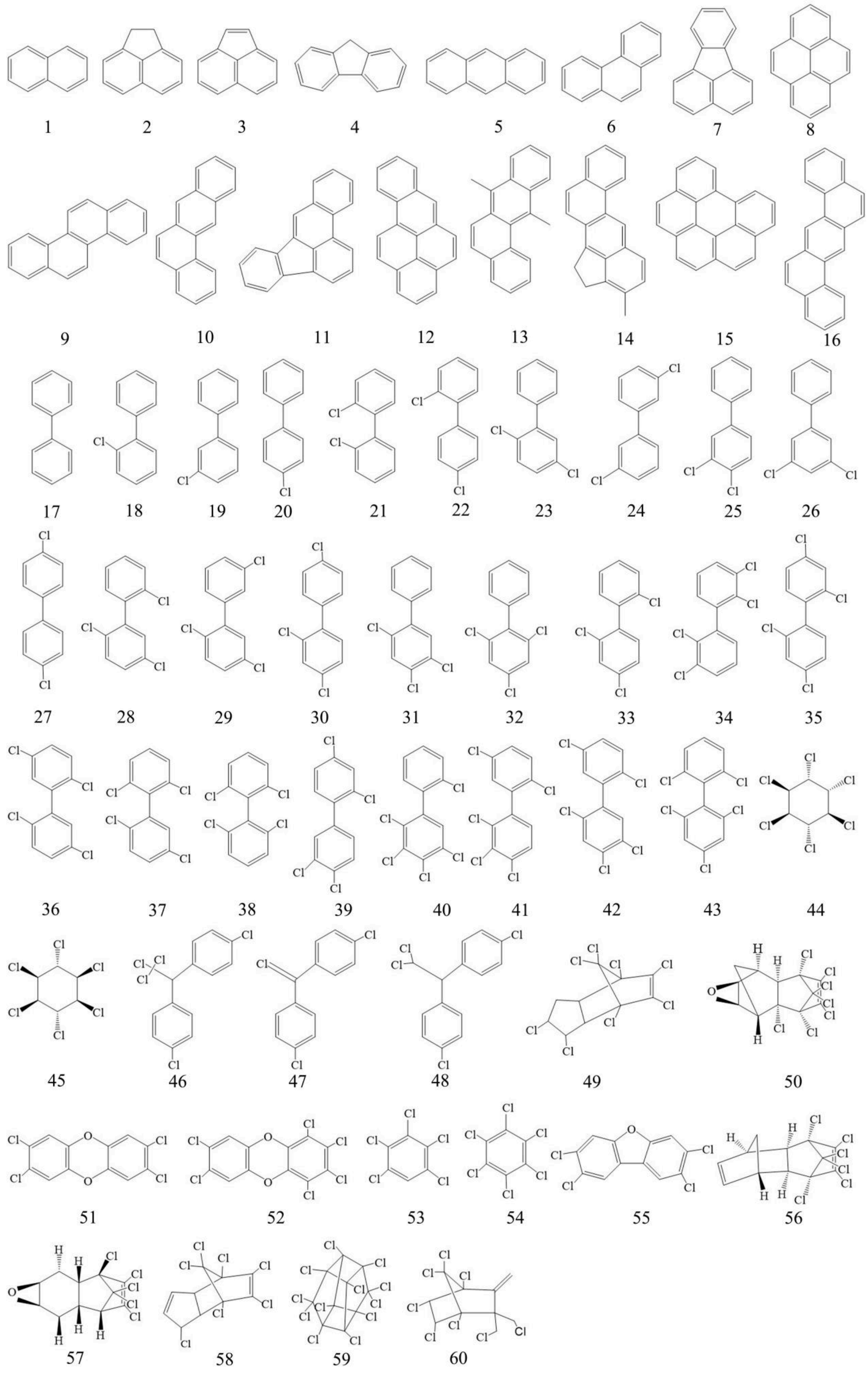

Figure S2 List several kinds of molecular fingerprints used in this study.

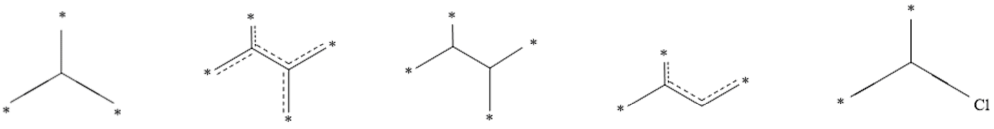

EPFC_1 6 | EPFC_1 5953 | EPFC_1 5961 | EPFC_1 5964 | EPFC_1 6294

Table S1 Features of principal component analysis.

Principal component number	Eigenvalue	Percentage of variance	Cumulative
1	2.83	40.42	40.42
2	2.11	30.20	70.62

Table S2 The correlation matrix of the five descriptors used in this study.

	HOMO_Energy_DMol3	Dipole_Z	SAscore_Fragments	SC_3_P	SIC
HOMO_Energy_DMol3	1.0	0.20	0.39	-0.079	0.039
Dipole_Z	0.20	1.0	0.58	-0.27	0.24
SAscore_Fragments	0.39	0.58	1.0	-0.73	0.032
SC_3_P	-0.079	-0.27	-0.73	1.0	-0.063
SIC	0.039	0.24	0.032	-0.063	1.0

Table S3 Prediction of additional POPs by MLR modeling.

Name	IUPAC name	Exp	Pred
PCB 6	2,3'-Dichlorobiphenyl	1.720	1.762
PCB 16	2,2',3-Trichlorobiphenyl	1.560	1.630
PCB 19	2,2',6-Trichlorobiphenyl	1.920	1.935
PCB 25	2,3',4-Trichlorobiphenyl	2.010	2.112
PCB 32	2,4',6-Trichlorobiphenyl	2.050	2.037
PCB 42	2,2',3,4'-Tetrachlorobiphenyl	2.150	2.135
PCB 43	2,2',3,5-Tetrachlorobiphenyl	2.270	2.055
PCB 44	2,2',3,5'-Tetrachlorobiphenyl	2.240	2.252
PCB 45	2,2',3,6-Tetrachlorobiphenyl	2.080	1.949
PCB 46	2,2',3,6'-Tetrachlorobiphenyl	2.030	2.007

Table S4 MLR model parameters for additional POPs.

**MODEL**	R^2	R^2_{text}	R^2_{adj}	Q^2_{cv}	$RMSEt_{est}$	MAE_{test}	*RE(%)*
MLR	0.919	0.828	0.900	0.749	0.091	0.064	3.156
